# Supplementary material for: Design and Accuracy of an Instrumented Insole Using Pressure Sensors for Step Count
Source: Sensors (Basel). 2019 Feb 26;19(5):984. doi: 10.3390/s19050984 (PMC6427154; doi:10.3390/s19050984)
Supplement: Supplementary file 1 [file sensors-19-00984-s001.pdf]

Supplementary Materials

Table S1. Accuracies for step count using instrumented insole, and GaitUp compared with manual counting at self-selected speed (1.43 ± 0.18 m/s)[a] in indoor

Participants	Individual FSR					Combined five FSRs		GaitUp
	FSR_H	FSR_{M5}	FSR_{M3}	FSR_{M1}	FSR_T	Average	Cumulative sum	
1	98.7%	96.5%	91.7%	68.5%	91.4%	96.2%	100.0%	100.0%
2	99.7%	100.0%	97.8%	99.1%	95.6%	99.1%	100.0%	99.7%
3	97.5%	100.0%	97.2%	99.3%	97.9%	98.9%	97.9%	100.0%
4	95.3%	99.1%	95.6%	96.8%	99.7%	96.2%	100.0%	99.7%
5	100.0%	100.0%	99.8%	99.8%	99.8%	95.5%	99.5%	99.3%
6	100.0%	92.6%	99.4%	83.9%	92.0%	93.5%	100.0%	100.0%
7	93.5%	99.7%	88.7%	100.0%	98.6%	94.4%	99.7%	100.0%
8	100.0%	95.3%	99.2%	96.1%	95.8%	97.4%	100.0%	100.0%
9	99.5%	100.0%	100.0%	99.5%	99.0%	94.2%	98.7%	99.7%
10	100.0%	95.6%	96.8%	95.6%	93.0%	99.1%	100.0%	NA
11	98.8%	100.0%	98.8%	99.7%	99.7%	86.2%	100.0%	100.0%
12	98.6%	97.8%	98.6%	98.9%	97.8%	94.8%	98.6%	100.0%
**Mean±SD**	98.5%±2.1%	98.1%±2.5%	97.0%±3.5%	94.8%±9.4%	96.7%±3.1%	95.5%±3.5%	99.5%±0.7%	99.9%±0.2%

a: Walking speeds were measured with GaitUp ; NA: Non Available.

Table S2. Accuracies for step count using instrumented insole, and GaitUp compared with manual counting at maximal speed (1.73 ± 0.08 m/s)[a] in indoor

Participants	Individual FSR					Combined five FSRs		GaitUp
	FSR_H	FSR_{M5}	FSR_{M3}	FSR_{M1}	FSR_T	Average	Cumulative sum	
1	98.7%	99.7%	99.2%	99.0%	99.0%	99.2%	100.0%	100.0%
2	98.9%	98.9%	99.2%	98.7%	100.0%	99.7%	99.7%	99.7%
3	97.9%	99.2%	97.6%	98.7%	99.5%	99.7%	100.0%	100.0%
4	98.1%	98.8%	98.6%	99.8%	99.3%	99.8%	99.3%	100.0%
5	99.8%	99.8%	99.8%	99.8%	99.5%	99.5%	99.1%	100.0%
6	99.7%	99.2%	99.7%	97.7%	98.7%	95.3%	100.0%	100.0%
7	98.7%	98.7%	99.7%	99.0%	98.2%	98.2%	99.0%	99.5%
8	98.1%	98.8%	98.3%	98.8%	98.5%	98.8%	99.0%	99.5%
9	95.9%	99.0%	95.3%	95.3%	98.8%	100.0%	99.8%	99.8%
10	96.9%	97.1%	90.6%	99.3%	95.4%	99.0%	99.8%	99.5%
11	99.7%	99.0%	99.7%	97.7%	97.1%	99.0%	100.0%	99.7%
12	99.2%	99.5%	99.2%	99.2%	99.5%	92.8%	99.2%	100.0%
**Mean±SD**	98.4%±1.2%	99.0%±0.7%	98.1%±2.7%	98.6%±1.2%	98.6%±1.3%	98.6%±2.2%	99.6%±0.4%	99.8%±0.2%

a: Walking speeds were measured with GaitUp.

Table S3. Accuracies for step count using instrumented insole, and GaitUp compared with manual counting at self-selected speed (1.45 ± 0.20 m/s)[a] in outdoor

Participants	Individual FSR					Combined five FSRs		GaitUp
	FSR_H	FSR_{M5}	FSR_{M3}	FSR_{M1}	FSR_T	Average	Cumulative sum	
1	99.4%	99,7%	99,7%	98,8%	98,8%	99.1%	99.7%	100.0%
2	98.9%	100,0%	99,7%	99,5%	99,7%	99.7%	100.0%	100.0%
3	98.0%	98,2%	98,2%	99,1%	99,7%	99.7%	98.8%	99.4%
4	98.3%	100,0%	100,0%	99,7%	99,3%	99.1%	100.0%	99.8%
5	99.7%	99,5%	99,0%	99,2%	99,2%	96.9%	99.2%	99.7%
6	99.1%	83,6%	97,0%	80,5%	82,7%	95.4%	99.7%	99.1%
7	100.0%	97,8%	96,1%	99,7%	98,6%	96.1%	99.4%	99.7%
8	92.1%	95,0%	92,4%	95,8%	95,0%	86.4%	100.0%	100.0%
9	95.9%	99,1%	97,7%	99,1%	97,7%	94.7%	99.1%	99.7%
10	99.4%	99,4%	94,9%	98,4%	97,8%	99.0%	100.0%	100.0%
11	96.1%	89,5%	95,5%	91,6%	93,1%	92.8%	99.1%	100.0%
12	99.5%	99,2%	98,9%	98,9%	98,1%	98.6%	99.5%	100.0%
**Mean±SD**	98.0±2.3%	96.7±5.1%	97.4±2.3%	96.7±5.6%	96.6±4.8%	96.5±3.9%	99.5±0.4%	99.8±0.3%

a: Walking speeds were measured with GaitUp.

Table S4. Accuracies for step count using instrumented insole, and GaitUp compared with manual counting at maximal speed (1.78 ± 0.12 m/s)[a] in outdoor

Participants	Individual FSR					Combined five FSRs		GaitUp
	FSR_H	FSR_{M5}	FSR_{M3}	FSR_{M1}	FSR_T	Average	Cumulative sum	
1	100.0%	99.5%	100.0%	99.5%	99.7%	98.0%	99.7%	99.7%
2	99.8%	99.0%	98.3%	99.8%	99.8%	98.3%	99.8%	99.8%
3	98.7%	99.7%	99.0%	99.7%	100.0%	97.4%	99.0%	100.0%
4	100.0%	96.7%	97.6%	97.6%	97.6%	96.2%	100.0%	99.8%
5	99.8%	99.1%	99.8%	99.1%	99.3%	98.0%	99.3%	100.0%
6	99.5%	99.0%	99.5%	98.2%	98.7%	92.4%	98.7%	99.5%
7	100.0%	99.8%	99.8%	99.8%	100.0%	99.0%	100.0%	99.5%
8	99.8%	97.6%	99.5%	98.3%	99.5%	98.5%	99.3%	99.8%
9	99.8%	96.0%	97.8%	97.2%	100.0%	87.5%	100.0%	99.8%
10	95.9%	77.7%	96.6%	99.0%	98.8%	99.8%	99.5%	100.0%
11	99.7%	99.7%	100.0%	99.7%	99.7%	99.2%	100.0%	100.0%
12	99.7%	99.7%	99.7%	99.7%	99.7%	99.5%	99.7%	100.0%
**Mean±SD**	99.4±1.2%	97.0±6.2%	99.0±1.1%	99.0±0.9%	99.4±0.7%	97.0±3.6%	99.6±0.4%	99.8±0.2%

a: Walking speeds were measured with GaitUp.
